# Long-term follow-up of intraocular pressure and pressure-lowering medication in patients following Excimer laser trabeculotomy

**DOI:** 10.1007/s00417-020-05029-4

**Published:** 2020-12-08

**Authors:** C. Deubel, D. Böhringer, A. Anton, T. Reinhard, J. Lübke

**Affiliations:** 1 Eye Center, Medical Center – University of Freiburg, Killianstrasse 5, 79106 Freiburg, Germany; 2 Faculty of Medicine, University of Freiburg, Freiburg, Germany

**Keywords:** ELT, Excimer laser trabeculotomy, Glaucoma, MIGS

## Abstract

**Background:**

Excimer laser trabeculotomy (ELT) is a minimally invasive procedure to lower the intraocular pressure (IOP) via a photo-ablative laser that is applied to the trabecular meshwork. With this procedure, it is possible to improve the outflow of the aqueous humor. Until now, a limited number of studies examining mostly relatively small sample sizes with midterm follow-up exist. We therefore present the analysis of a large ELT cohort in a long-term follow-up.

**Methods:**

We recorded data from 580 patients who underwent ELT or combined ELT with cataract surgery at our institution from November 2000 until March 2011. A total of 512 patients with primary open angle glaucoma (POAG), pseudoexfoliation glaucoma (PEX), and ocular hypertension (OHT) were included in the analysis. At every follow-up examination, the usage of IOP-lowering medication and the IOP were recorded. Failure criteria were defined as the need for another surgical glaucoma procedure, when the IOP was not 21 mmHg or less and a reduction of 20% from the baseline was not achieved with (qualified success) or without (absolute success) additional medication. Statistical analysis was done using Kaplan-Meier analysis and Cox regression.

**Results:**

Four hundred twenty-eight patients underwent combined cataract and ELT surgery, and 84 underwent solitary ELT surgery. After a median follow-up time of 656 days, 87% (combined surgery) and 66% (ELT) of the patients did not have to undergo another IOP-lowering intervention; 47/31% were classified as a qualified success and 31/11% as a complete success. The IOP-lowering medication, however, could not be significantly reduced within that time period.

**Conclusion:**

Especially when combined with cataract surgery, ELT is a feasible minimally invasive procedure to lower the IOP on a mid- to long-term basis. Over the long term, however, IOP-lowering medication could not be reduced.

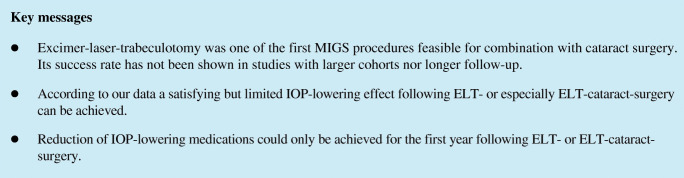



## Introduction

Glaucoma is one of the leading causes of blindness worldwide. Approximately 3.5% of the world’s population are affected by this eye disease [1]. It is a chronic disease that results in a progressive irreversible loss of retinal ganglion cells including their axons [2, 3]. It is empirically proven that the highest risk factor for developing glaucoma or its progression is an elevated IOP [3]. Furthermore, the only effective treatment for glaucoma is to lower the IOP [4, 5].

ELT is a laser-based minimally invasive glaucoma surgery performed by a photo-ablative laser targeting the trabecular meshwork [5]. The trabecular meshwork is one of the main outflow barriers, and the treatment increases the outflow of the aqueous humor [1]. ELT is usually performed via clear cornea incisions and therefore can be combined with cataract surgery.

Among the past studies investigating ELT, only smaller cohorts with a short to medium period of follow-up were included [5–8]. Herein, we wanted to evaluate and obtain a deeper insight into mid- to long-term treatment effects and analyzed a larger cohort with longer follow-up.

## Methods

The local ethics committee approved this study (vote no. 233/19). We identified all patients who underwent ELT surgery or ELT surgery combined with cataract surgery at the eye center in Freiburg, Germany, from November 2000 until March 2011. All medical reports dating until August 2019 were reviewed. Glaucoma surgeries, IOP values, and the number of prescribed IOP-lowering medications were extracted into a relational database for statistical analysis.

Only patients with primary open angle glaucoma (POAG), pseudoexfoliative glaucoma (PEX), and ocular hypertension (OHT) were included.

ELT was performed using the XeCl Excimer Laser AIDA via clear cornea incisions [4]. Acetylcholine was injected, or pilocarpine 2% eye drops were used to get a better sight of the trabecular meshwork. The laser treatment was performed under viscoelastic stabilization of the anterior chamber. Ten laser effects mostly within the lower circumference were applied [5]. In combination with cataract surgery, the phacoemulsification and posterior chamber lens implantation were performed prior the laser ablation.

Using the recommendations of the World Glaucoma Association, the surgical success was defined as “complete” or “qualified” [9]. The treatment was defined as a complete success if IOP was ≤ 21 mmHg, the IOP was lowered by at least 20%, and no further IOP-lowering medication compared to preoperatively was needed. In case of a qualified success, additional IOP-lowering medication was used. We performed time to event analyses. Endpoints were either time to first further glaucoma surgery in the study eye, time to the first IOP rise IOP above 80% of the preoperative IOP levels without topical glaucoma treatment (absolute success) or regardless of topical treatment (relative success). IOP-related events were not counted during the first 30 postoperative days. The postoperative median IOP was calculated as the median of all obtained postoperative IOP values. Failure criteria were defined as the need for another IOP-lowering procedure or an IOP elevation above the preoperative value.

Kaplan-Meier analysis and Cox regression were used for statistical inference. All data processing and analysis were done using the R system [10].

All analyses were performed for the group of ELT surgery as a standalone procedure and the group of combined surgery.

## Results

Descriptive data of the cohort are shown in Table 1. As a result, a total number of 512 eyes were included. Eighty-four surgeries were performed as a single procedure, and 428 surgeries were combined with cataract surgery. The median follow-up time after surgery was 656 days (approximately 1 year and 10 months). Four percent of the patients suffered from OHT, 29% from PEX, and 66% from POAG. The median age was 75.0 years, and 66% of the patients were female.

**Table 1:** Descriptive data including medians and quartiles

Total number included	512
Single ELT	84
Combined procedure	428
POAG	340 (66.3%)
PEX	149 (29.3%)
OHT	23 (4.3%)
Age (years)	75.0
1st quartile	69.0
3rd quartile	80.0
Female (%)	66
IOP preoperative (mmHg)	25.0
1st quartile	21.0
3rd quartile	28.0
IOP postoperative (mmHg)	17.0
1st quartile	14.8
3rd quartile	19.7
Follow-up time (days)	655.8
1st quartile	115.8
3rd quartile	1578.0

Combined procedure: ELT and cataract surgery

*POAG* primary open angle glaucoma, *PEX* pseudoexfoliative glaucoma, *OHT* ocular hypertension, *IOP* intraocular pressure

The median IOP for both groups preoperatively was 25.0 mmHg and 17.0 mmHg postoperatively.

Cox regression did not show a significant relation between diagnoses (POAG, PEX, and OHT), sex and age, and the outcome of ELT.

**Fig. 1:**
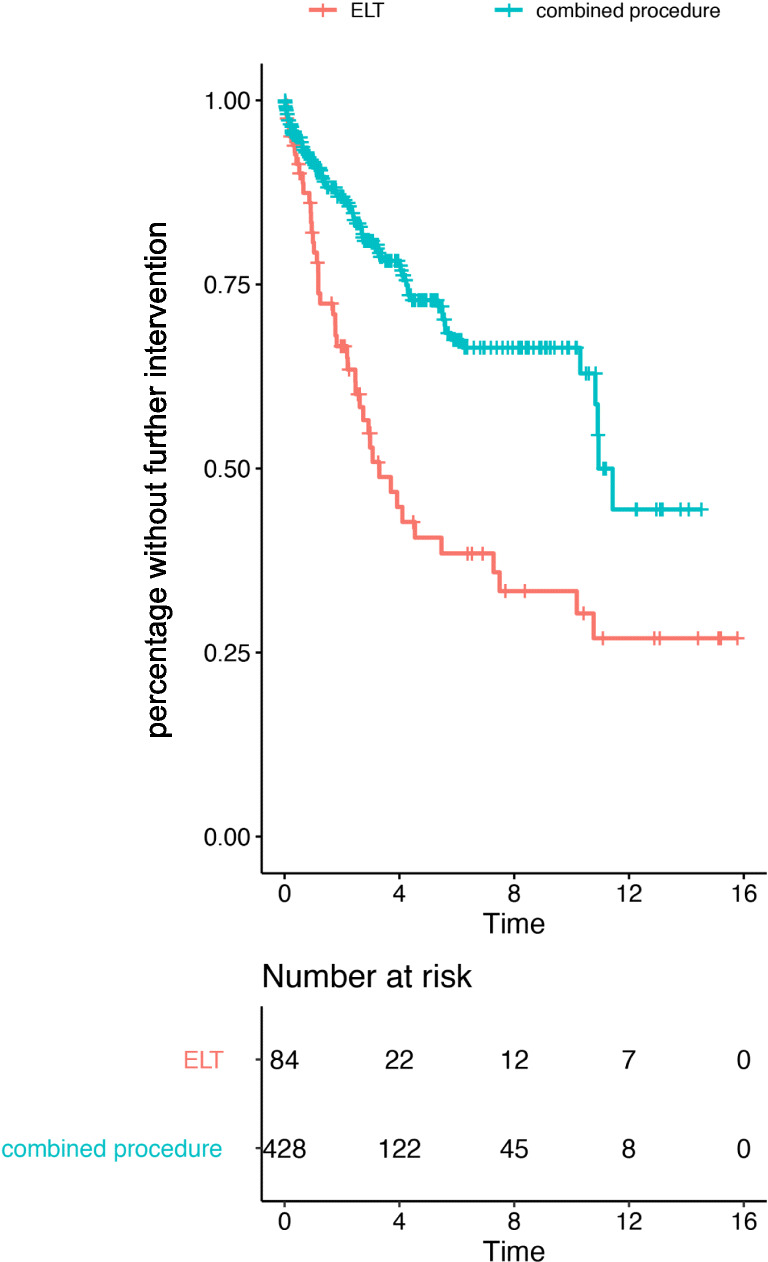
Kaplan-Meier analysis for the need of another intraocular pressure-lowering intervention

After the median follow-up, 87% of the patients in the combined surgery group did not need another IOP-lowering surgery, while it was 66% in the solitary ELT group. Kaplan-Meier analysis is shown in Fig. 1.

**Fig. 2:**
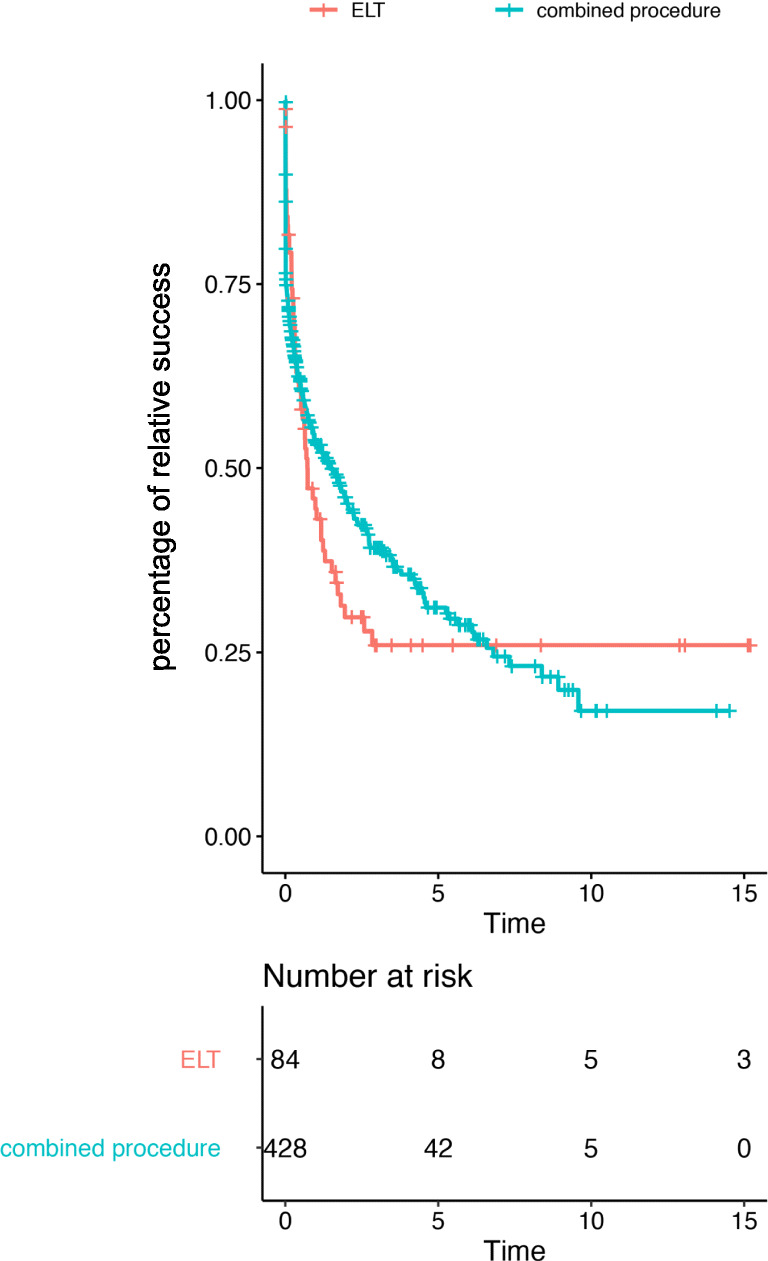
Kaplan-Meier analysis for qualified success following Excimer laser trabeculotomy

Figure 2 shows the Kaplan-Meier analysis for qualified success after ELT. After the median follow-up time, the qualified success was 47% for combined surgery and 31% for ELT alone.

**Fig. 3:**
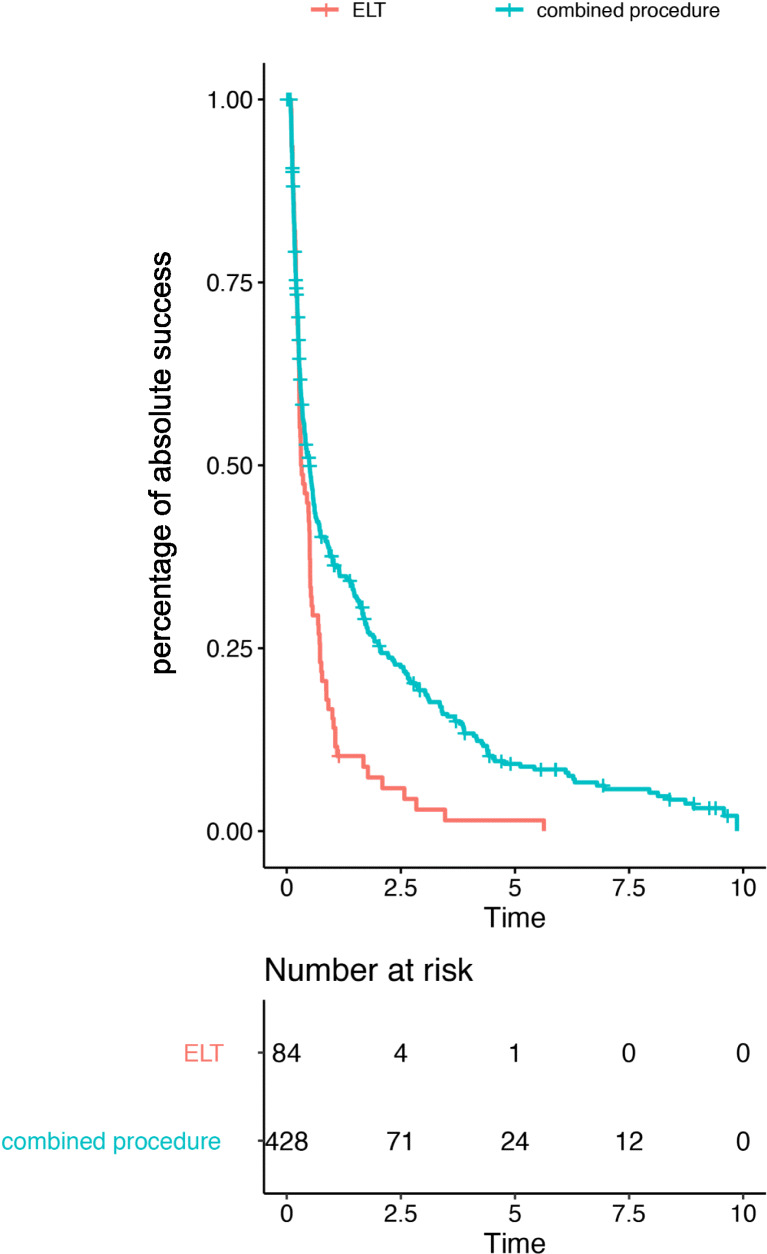
Kaplan-Meier analysis for complete success of Excimer laser trabeculotomy

Figure 3 shows the complete success following ELT. After the median follow-up of 656 days, the complete success rate was 31% and 11% for ELT alone.

**Fig. 4:**
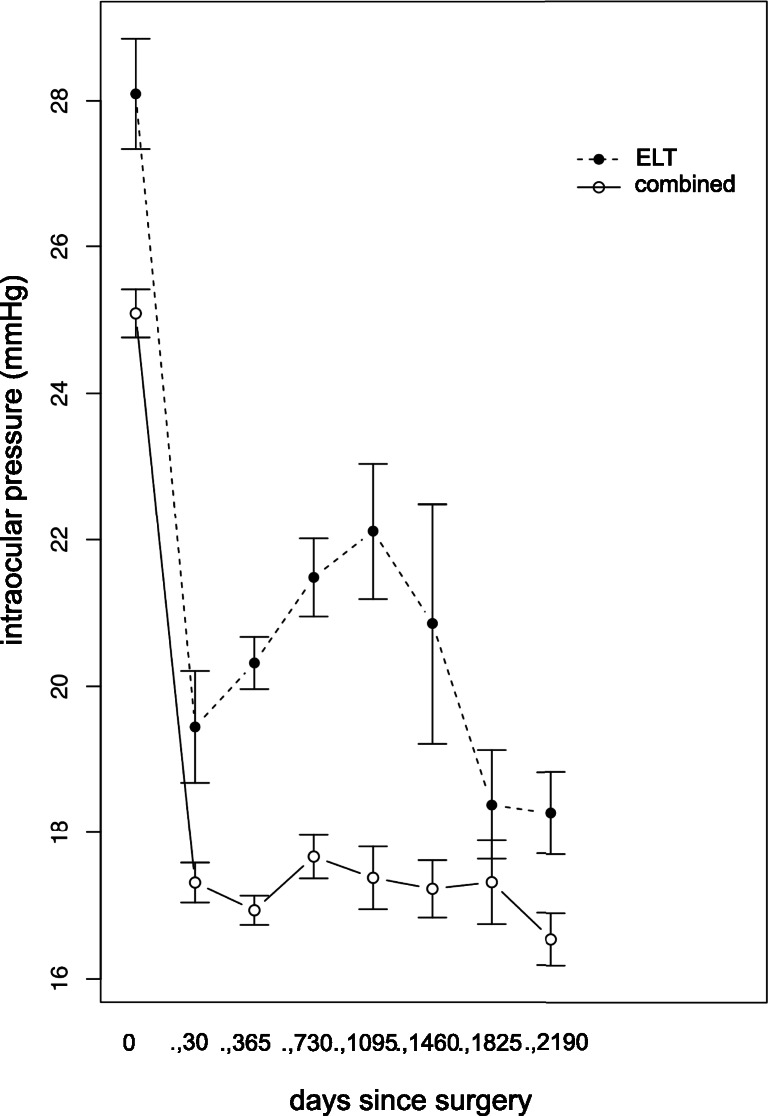
Mean intraocular pressure reduction

Mean IOP for the different postoperative time periods are shown in Fig. 4. Mean IOP reduction was almost 30% after the first year and nearly 24% after 2 years for ELT alone. We saw an increase of IOP during the following follow-up period until 3 years again. For combined surgery, the IOP could be reduced by 32% after 1 year and 30% after 2 years. IOP values showed a more stable reduction compared to ELT alone.

**Fig. 5:**
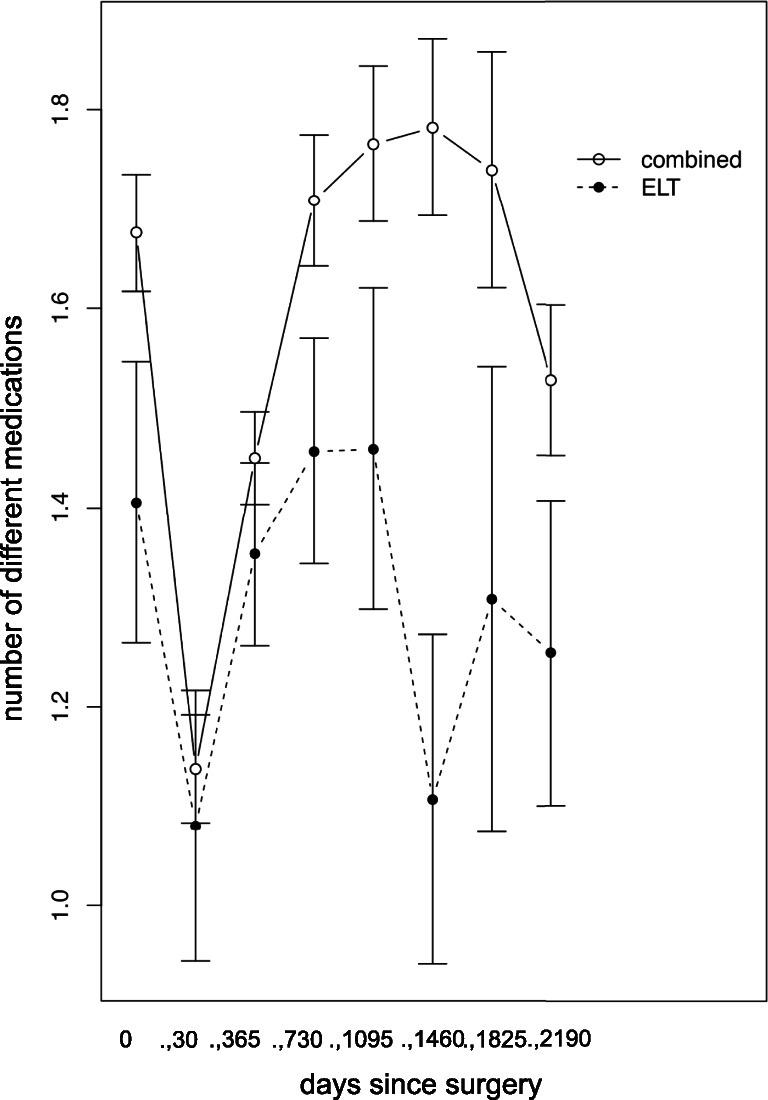
Number of intraocular pressure-lowering medications

After the first postoperative time period of 30 days, a reduction in IOP-lowering medication from 1.68 to less than 1.10 could be found for combined surgery. After the second time period, from 30 days until 1 year, the number of needed IOP-lowering medication increased again to 1.45. After 2 years, medication was approximately at the same level as preoperatively. For the group of ELT alone, IOP-lowering medication could be reduced from 1.45 to 1.08 within the first time period but rose again to almost the same or even more medication during the following time periods. IOP-lowering medication over the time is shown in Fig. 5.

## Discussion

The aim of this study was to analyze the long-term efficacy of ELT concerning the IOP-lowering effect and reduction of glaucoma medication. To our knowledge, there are only studies with shorter follow-ups or smaller cohorts of patients.

The Kaplan-Meier analysis shows that ELT could achieve partly satisfactory results. After a median follow-up of 656 days, 87%/66% of patients did not need another IOP-lowering intervention.

The IOP could be lowered by almost 30% within a period of 1 year postoperatively from 25.50 to less than 18.00 mmHg. IOP-lowering medication could be reduced for the first year after surgery but increased again after 2 years of follow-up.

In 2011, Töteberg-Harms et al. presented a study with a cohort of 24 eyes over a follow-up time of 12 months [11]. All of the patients received the ELT combined with cataract extraction. In this study, phaco-ELT could reduce the IOP by 8.79 mmHg ± 5.28 mmHg (− 34.70%). The average number of the IOP-lowering medication could be reduced by 0.79 ± 1.50 (− 62.70%).

In 2013, Töteberg-Harms et al. presented another study concerning the effect of phaco-ELT on IOP with a follow-up of 12 months [6]. Sixty-four eyes were included, and patients were divided into two groups based on preoperative IOP. The IOP of the group with lower preoperative IOP (16.5 ± 2.9 mmHg) could be lowered by 1.9 mmHg ± 4.4 mmHg (− 11.05%), and the average number of IOP-lowering medications could be reduced by 1.1 ± 1.4 (− 42.9%). Compared to this, the IOP in the group with higher preoperative IOP (25.8 ± 2.9 mmHg) could be lowered by 9.5 mmHg ± 5.4 mmHg, whereas the number of IOP-lowering medications could be reduced by 0.7 ± 1.6 (− 29.5%).

Babighian et al. presented a study comparing ELT to SLT (30 eyes) in 2010 [8]. The authors reported a complete success rate of 53.3% with ELT and 40% with SLT after 24 months. The IOP decreased by performing ELT from 25.0 ± 1.9 to 17.6 ± 2.2 mmHg (29.6%) and from performing SLT 23.9 ± 0.9 to 19.1 ± 1.8 mmHg (21%). However, the preoperative IOP in the SLT was lower than in the ELT group. Previous results, for example, in the Töteberg-Harms et al. study presented earlier, showed a significantly higher reduction of the IOP by ELT when IOP is elevated [6].

An ELT study from Wilmsmeyer et al. published in 2005 confirmed an IOP reduction within a cohort of 75 eyes [12]. After a follow-up at 2 to 4 months, and a reduction from 24.1 to 18.8 mmHg, the findings were reported as a qualified success of 60%.

Pache et al. (135 eyes) reported a 57% qualified success rate by patients with a preoperative IOP of > 22 mmHg and 41% by patients with a preoperative IOP of ≤ 22 mmHg after a follow-up of 12 months [7].

The aforementioned studies are consistent with the results from our study regarding the reduction of IOP, the success rates, and the number of IOP-lowering medication. Our data show a reduction of the IOP level of almost 30% within 1 year. ELT reduced the IOP from 25.50 to 18.00 mmHg, which is similar to the results of other studies that presented a 12-month follow-up. The number of IOP-lowering medication was reduced by almost 35% within the first 30 days after the surgery. After this period of time, the prescribed number of IOP-lowering medication increased again to preoperative levels. The success rate in our study shows a qualified success rate after 12 months follow-up of approximately 55/37% and a cumulative success rate of 37/23%.

Table 2 summarizes these ELT studies and our study.

**Table 2:** Comparison of our study to different studies analyzing Excimer laser trabeculotomy

Author/year	Patients included	Follow-up time	IOP reduction	Reduction of IOP-lowering medication
Töteberg-Harms et al. (2011)	24	12 months	34.70%	62.70%
Töteberg-Harms et al. (2013)	64	12 months	Higher IOP: 36.82% Lower IOP: 11.05%	Higher IOP: 29.50% Lower IOP: 42.90%
Babighian et al. (2010)	30	24 months	29.60%	–
Wilmsmeyer at al. (2005)	75	2–4 months	21.99%	–
Current study (2020)	512	12 months 21 months	32.00/30.00% 30.00/24.00%	14.00/4.00% 0/0%

*IOP* intraocular pressure

In comparison to other less invasive laser-based IOP-lowering procedures that target the outflow through the trabecular meshwork, our data suggest a higher success rate.

In 2017, Conlon et al. presented a review concerning the effectiveness of selective laser trabeculoplasty (SLT) [13]. The authors show a mean “survival” of approximately 2 years. At 12 months, the SLT reduces the IOP at least 20% below baseline level in 58–94% of the patients, after 2 years in 40–85%. The results of SLT compared to ALT are almost identical as shown in Wand et al. [14]*.*

Our data show a lower failure rate after ELT compared to studies on SLT and ALT. Nevertheless, comparing the SLT or ALT to the ELT, one has to mention that the ELT is more invasive and has to be performed as an intraocular surgery. The SLT or ALT can be performed as a slit lamp procedure without opening the bulb. Therefore, the risks of serious complications are higher performing ELT than SLT or ALT.

As another intraocular surgical approach to improve the outflow of the aqueous humor, a comparison with trabectome surgery might be interesting. The three following studies obtain a similar cohort since they are from the same university hospital. They differentiate within follow-up time and number of patients.

Most recently in 2019, Avar et al. published a study confirming an IOP-lowering effect of the trabectome within 3.5 years from 23.0 ± 5.8 to 16.5 ± 4.1 mmHg [1]. The number of IOP-lowering medication in POAG/PEX could be reduced from 2.8/2.4 to 1.9/1.7.

A qualified success of 44.6%/67.5% (POAG/PEX) was reached. In our study, the ELT showed a similar outcome. After 3.5 years, the IOP could be reduced from 25.50 to 18.00 mmHg, and the qualified success rate was 40/27%. The number of IOP-lowering medication though could not be reduced in our data like in the Avar et al. study [1]. After a 3.5 year follow-up, the IOP-lowering medication after ELT increased again to its preoperative level.

Another trabectome study published in 2011 by *Jordan* et al. showed an IOP reduction after 313 days from 25.0 to 17.0 mmHg and a reduction of the IOP-lowering medication from 2.0 to 1.5 [15]. Our results show an IOP reduction after 313 days from 25.50 to 17.50 mmHg, and within this period of time, the ELT could reduce the number of IOP-lowering medication from 1.68 to 1.45 (combined surgery) and 1.45 to 1.38 (ELT).

Wecker et al. presented in 2017 in their retrospective study a reduction through trabeculotomy from 25.2 to 16.3 mmHg after 125 days [14]. The number of IOP-lowering medication was reduced from 2.14 to 1.50. In comparison with the data presented, the IOP was reduced from 25.50 to 17.50 mmHg, and the reduction of the IOP-lowering medication was from 1.68 to 1.30 (combined) and 1.45 to 1.20 (ELT) after 125 days.

Both procedures, ELT and trabeculotomy, are similar in invasiveness and technique. They do not interfere with invasive filtration surgery and can be combined while performing cataract surgery.

In summary, our data confirm the results reported in the previous ELT studies for a longer follow-up and a larger group of patients. ELT may give comparable results to trabectome surgery as another minimal invasive glaucoma surgery technique that targets the trabecular meshwork and its outflow capability. Compared to other trabecular meshwork laser procedures (SLT and ALT), ELT may confer better long-term outcomes.

Nevertheless ELT is still a limited procedure especially in the long-term and does not deliver sustainable IOP-lowering as more invasive procedures like filtration surgery.

## Limitation of this study

First of all, the chosen study design limits the outcome since a retrospective study increases the risk of systemic bias. It must be considered that a lot of successful ELT interventions might not be included long-term-wise in this study since the follow-up examinations of uncomplicated cases might have been done by ophthalmologists in private practice.

## Conclusion

In conclusion, the ELT used solitary or in combination with cataract extraction is a feasible method to lower the IOP. In midterm, the ELT shows IOP reduction especially when combined with cataract surgery, but a long-term reduction of the number of IOP-lowering medication does not seem achievable.

## Data Availability

Data is available on reasonable request.
